# Correlations Between Plasma BNP Level and Risk of Thrombotic-Hemorrhagic Events After Left Atrial Appendage Closure

**DOI:** 10.3390/jcm13206232

**Published:** 2024-10-18

**Authors:** Teruhiko Imamura, Naoya Kataoka, Shuhei Tanaka, Hiroshi Ueno, Koichiro Kinugawa, Masaki Nakashima, Masanori Yamamoto, Mitsuru Sago, Ryuki Chatani, Masahiko Asami, Daisuke Hachinohe, Toru Naganuma, Yohei Ohno, Tomoyuki Tani, Hideharu Okamatsu, Kazuki Mizutani, Yusuke Watanabe, Masaki Izumo, Mike Saji, Shingo Mizuno, Shunsuke Kubo, Shinichi Shirai, Kentaro Hayashida

**Affiliations:** 1 Second Department of Internal Medicine, University of Toyama, Toyama 930-0194, Japan; nkataoka@icloud.com (N.K.);; 2 Department of Cardiology, Sendai Kousei Hospital, Sendai 980-0873, Japan; 3 Department of Cardiology, Toyohashi Heart Center, Toyohashi 441-8071, Japan; 4 Department of Cardiology, Nagoya Heart Center, Nagoya 461-0045, Japan; 5 Department of Cardiology, Gifu Heart Center, Gifu 500-8384, Japan; 6 Department of Cardiology, Kurashiki Central Hospital, Kurashiki 710-0052, Japan; rc15756@kchnet.or.jp (R.C.);; 7 Division of Cardiology, Mitsui Memorial Hospital, Tokyo 101-8643, Japan; 8 Department of Cardiology, Sapporo Heart Center, Sapporo Cardio Vascular Clinic, Sapporo 007-0849, Japan; 9 Department of Cardiology, New Tokyo Hospital, Chiba 270-2232, Japan; 10 Department of Cardiovascular Medicine, Graduate School of Medical Sciences, Kumamoto University, Kumamoto 860-0811, Japan; 11 Department of Cardiology, Tokai University School of Medicine, Kanagawa 247-8533, Japan; 12 Department of Cardiology, Sapporo East Tokushukai Hospital, Sapporo 065-0033, Japan; 13 Department of Cardiology, Saiseikai Kumamoto Hospital, Kumamoto 860-0811, Japan; 14 Division of Cardiology, Department of Medicine, Kindai University Faculty of Medicine, Osaka-Sayama 589-8511, Japan; 15 Department of Cardiology, Teikyo University School of Medicine, Tokyo 173-0003, Japan; 16 Department of Cardiology, St. Marianna University School of Medicine, Kawasaki 216-8511, Japan; 17 Department of Cardiology, Sakakibara Heart Institute, Tokyo 183-0003, Japan; 18 Department of Cardiology, Shonan Kamakura General Hospital, Kanagawa 247-8533, Japan; 19 Department of Cardiology, Kokura Memorial Hospital, Fukuoka 802-8555, Japan; 20 Department of Cardiology, Keio University School of Medicine, Tokyo 160-8582, Japan

**Keywords:** heart failure, stroke, bleeding, WATCHMAN

## Abstract

**Background:** Percutaneous left atrial appendage closure (LAAC) reduces the incidence of stroke/bleeding events in patients with non-valvular atrial fibrillation, high risk of stroke, and contraindication in continuing anticoagulation therapy. Of them, patients with heart failure may remain at high risk of these events after LAAC. **Method:** Patients who underwent LAAC and were listed for the multi-center, prospectively collected OCEAN-LAAC registry, were eligible. Of them, individuals without baseline plasma B-type natriuretic peptide (BNP) levels and those dependent on hemodialysis were excluded. The prognostic impact of baseline plasma BNP levels on the incidence of death or stroke/bleeding events after LAAC was evaluated. **Results:** A total of 937 patients (median 78 years, 596 men) were included. The LAAC device was successfully implanted in 934 (98%) patients. Over the 366 (251, 436) days after the LAAC, 148 patients encountered a primary outcome. The common logarithm of baseline plasma BNP was independently associated with the primary outcome with an adjusted hazard ratio of 1.46 (95% confidence interval 1.06–2.18, *p* = 0.043). A calculated cutoff of 2.12 (equivalent to 133 pg/mL of plasma BNP level) significantly stratified the cumulative incidence of the primary outcome (29% vs. 21% for 2 years, *p* = 0.004). **Conclusions:** Using prospectively collected large-scale multi-center Japanese registry data, we demonstrated that a baseline higher plasma BNP level was independently associated with a higher incidence of stroke/bleeding events and mortality after LAAC. Further studies are warranted to understand the optimal therapeutic strategy for LAAC candidates with elevated baseline plasma BNP levels.

## 1. Introduction

Atrial fibrillation is a common tachyarrhythmia worldwide and can manifest regardless of severe structural heart disease [1]. Amongst the most important considerations is the significantly higher cumulative risk of thromboembolic stroke in patients with atrial fibrillation compared to those in sinus rhythm [2]. Thus, it is of utmost importance to implement evidence-based therapeutic strategies to reduce the risk of atrial fibrillation-related stroke events [3].

Vitamin K antagonists are the classic oral anticoagulants (OACs) that reduce the risk of stroke and mortality in individuals with atrial fibrillation [4]. However, the drawbacks of this medication are drug-induced bleeding and the difficulty in maintaining therapeutic levels within the optimal ranges [5]. Furthermore, changes in diet, nutritional status, hepatic function, and drug interactions can all affect the international normalized ratio levels. Direct oral anticoagulants (DOACs) have become superior alternatives to conventional vitamin K antagonists in many clinical scenarios, given their superior impact on preventing stroke, ease of prescription [6], and lower risk of drug-induced bleeding [7]. Nevertheless, some patients cannot tolerate any continuous OAC therapies due to a history of bleeding, for which widely available reversal agents for DOACs are both very expensive and not always available [8,9].

Left atrial appendage closure (LAAC) is a recently innovated non-pharmacological alternative to OAC for individuals with non-valvular atrial fibrillation who have contraindications to continuous OAC therapy despite high risk for stroke [10]. Several randomized control trials demonstrated the non-inferiority of LAAC using the WATCHMAN device (Boston Scientific, Marlborough, MA, USA) in reducing the risk of stroke and bleeding versus vitamin K antagonist (PROTECT AF and PREVAIL) [11,12] or DOAC (PRAGUE-17) [12]. Given these findings, LAAC using the WATCHMAN device received FDA approval in 2015. Following the SALUTE trial [13,14], LAAC was reimbursed in Japan, in 2019. The PINNACLE FLX study further demonstrated the feasibility and efficacy of the next-generation WATCHMAN FLX [15].

However, some patients still encounter stroke/bleeding events following LAAC therapy. For example, patients with atrial fibrillation often have coexisting systolic heart failure. There is a strong dual relationship between atrial fibrillation and heart failure [16]. The presence of atrial fibrillation impairs cardiac output, whereas the presence of heart failure increases the burden on the left atrium. The imbalance of the autonomic nervous system facilitates the development of atrial fibrillation, together with the presence of cardiac ischemia and myocardial scar tissue [17]. The presence of atrial fibrillation further progresses atherosclerosis and cardiac demand, worsening cardiac ischemia [18].

Previous literature demonstrated a causal relationship between atrial fibrillation and an incremental risk of stroke and bleeding [19,20]. These findings were applied to subjects who underwent catheter ablation for atrial fibrillation, particularly in patients with heart failure and preserved ejection fraction [21]. The presence of heart failure may theoretically increase the risk of stroke/bleeding events even after LAAC, although few studies have confirmed this risk association. These data may further improve pre-procedural risk stratification, support appropriate post-LAAC monitoring, and allow for better informed decisions on peri-procedural antithrombotic medication.

Diagnoses of clinical heart failure can be challenging in patients with atrial fibrillation due to the potential overlap of concomitant symptoms between the two conditions, such as dyspnea on effort and palpitation [22]. The measurement of plasma B-type natriuretic peptide (BNP) levels, as an objective and continuous surrogate of heart failure severity, allows us to assess the severity of heart failure. In the present study, we evaluated the impact of baseline plasma BNP level, as an objective indicator alternative to diagnosing heart failure, on the incidence of stroke/bleeding events after LAAC, using the Japanese large-scale multi-center optimized catheter valvular intervention (OCEAN)-LAAC registry [23].

## 2. Methods

### 2.1. OCEAN-LAAC Registry

The OCEAN-LAAC is an ongoing, prospective, investigator-initiated, multicenter observational registry that includes patients with non-valvular atrial fibrillation who underwent percutaneous LAAC in 20 Japanese centers (UMIN000038498) [23]. A list of investigators and their sites are displayed in Supplementary Table S1. The ethics committees of all participating centers approved the protocol of the registry. The registry management was conducted as per the Declaration of Helsinki. All participants provided informed consent for the intervention and registry listing before being enrolled in this registry.

The WATCHMAN 2.5 device was used until May 2021. Afterward, the WATCHMAN FLX device received clinical approval and the WATCHMAN 2.5 was replaced with this new generation device [24].

### 2.2. Patient Selection

**Figure 1:**
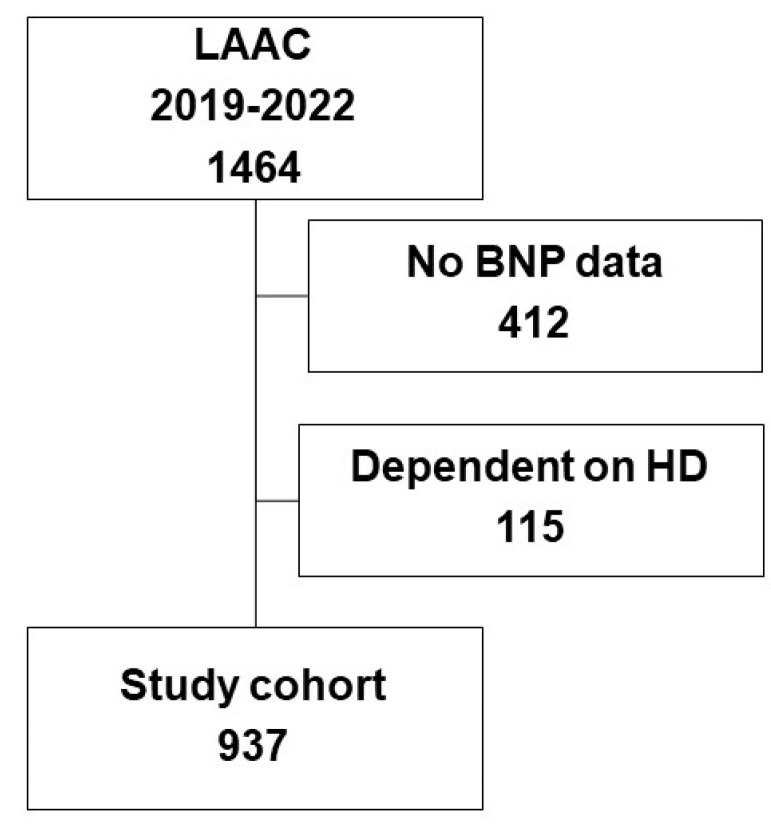
Flow chart of the patients’ inclusion. Patients who underwent LAAC and were listed in the OCEAN registry between 2019 and 2022 were eligible. Of them, patients without baseline plasma BNP levels and those dependent on HD were excluded. Finally, 937 patients were included. LAAC, left atrial appendage closure; BNP, B-type natriuretic peptide; HD, hemodialysis.

Data on patients who underwent LAAC between 2019 and 2022 and were listed on the registry were retrospectively used in this study. Of them, patients without baseline plasma BNP levels and those dependent on hemodialysis were excluded (Figure 1).

### 2.3. Study Design

The independent variable was defined as a baseline plasma BNP level, which was transformed into the common logarithm for statistics given their non-normal distribution. The primary outcome was defined as a composite of death and stroke/bleeding events after LAAC, consisting of ischemic stroke, systemic embolism, device-related thrombus, gastrointestinal bleeding, hemorrhagic stroke, and other major bleeding. The prognostic impact of an independent variable on the primary outcome was evaluated.

### 2.4. Indication of LAAC

Anticoagulation therapy is indicated in patients with atrial fibrillation and high risk for stroke based on CHADS_2_ score or CHA_2_DS_2_-VASc score. For them, LAAC was considered as an alternative to anticoagulation therapy when patients were at high risk for bleeding and satisfied one of the following items [25]: (1) HAS-BLED score equal to or above 3; (2) a history of multiple instances of trauma from falls requiring treatment; (3) a history of cerebral amyloid angiopathy; (4) the necessity of receiving a combination of dual-antiplatelet therapies (DAPT) for over a year; and (5) a history of major bleeding classified as Bleeding Academic Research Consortium type 3.

The exclusion criteria for LAAC were as follows: (1) thrombus in the atrium or appendage; (2) a history of surgical treatment for atrial septal defect or patent foramen ovale; (3) unsuitable morphology for LAAC using the WATCHMAN system; (4) contraindications for transesophageal echocardiography; (5) contraindications for anticoagulation therapy or any antiplatelet therapies; (6) active systemic infection or inflammation; and (7) no agreement to the proposed informed consent.

The final decision to perform LAAC was determined by the internal heart-valve team considering risk and benefit, together with careful informed consent from patients and their relatives.

### 2.5. LAAC Procedure

LAAC was performed under general anesthesia and angiographic/echocardiographic guidance via the femoral vein and trans-septal approach according to the standard procedure by the board-certificated interventionalists, who had completed company-specified physician training programs [25]. WATCHMAN 2.5 or WATCHMAN FLX were utilized in LAAC, with their sizes selected carefully to accommodate the anatomy and size of each LAA before the procedure.

### 2.6. Definition of Implantation Outcome

The implantation outcome was assessed using several definitions. The implantation success was defined by the precise deployment of the device in LAA with the appropriate position. The technical success was defined as a complete closure of the LAA with a peri-device leakage of less than 5 mm and the absence of device-related complications. The procedural success required the absence of any procedural-related complications; in addition to the assumption of technical success.

### 2.7. Post-Procedural Antithrombotic Therapy

According to the previous literature, it is recommended to add low-dose aspirin to OAC after LAAC. If the 45-day imaging documented that the depth of peri-device flow was <5 mm and there was no obvious device-related thrombosis, OAC was discontinued and clopidogrel was added. Finally, clopidogrel was discontinued 6 months after LAAC. In real-world practice, the regimen of antithrombotic therapy after LAAC was determined by each center given the lack of robust evidence and the variety of patients’ backgrounds.

### 2.8. Data Collection

Baseline characteristics just before LAAC were collected, including demographics, comorbidities, laboratory, echocardiography, and medication data. The risk of thromboembolic events was estimated by calculating baseline CHADS_2_ score and CHA_2_DS_2_-VASc scores. The risk of bleeding was estimated by calculating the baseline HAS-BLED score. Of note, the baseline plasma BNP level was defined as an independent variable.

Intra-procedural and peri-procedural data were collected, including device type, device size, and general procedure data. Peri-procedural complication data were also collected.

After index discharge, patients were followed at each center by board-certified cardiologists. Follow-up imaging was obtained at 45–90 days (short-term follow-up) and 1 year after LAAC, by utilizing trans-esophageal echocardiography, in principle. Device size, device compression, device positioning, and device-related thrombosis were investigated.

The primary outcome was defined as a composite of death and stroke/bleeding events, consisting of ischemic stroke, systemic embolism, device-related thrombus, gastrointestinal bleeding, hemorrhagic stroke, and other major bleeding. Major bleeding was defined as Bleeding Academic Research Consortium type 3 or 5.

### 2.9. Statistical Analyses

Statistical analyses were performed with SPSS Statistics 23 (SPSS Inc., Armonk, IL, USA). Two-sided *p*-values < 0.05 were considered statistically significant. Continuous variables were expressed as median (25% interquartile, 75% interquartile) and compared between the two groups by Mann–Whitney U test. Categorical variablees were stated as numbers and percentage and compared bewteen the two groups by Chi-square test or Fischer’s exact test.

The independent variable was defined as baseline plasma BNP level, which was converted to the common logarithm. The primary outcome (a dependent variable) was defined as a composite of death and stroke/bleeding events after LAAC, as detailed above. The prognostic impact of the independent variable on the primary outcome was evaluated by Cox proportional hazard ratio regression analysis, one of the most standard time-to-event analyses, by adjusting for other baseline characteristics, which were pre-specified before performing statistics as potential risk factors. These potential variables were included in the univariable analyses. Variables with *p* < 0.10 in the univariable analyses were included in the multivariable analysis as potential confounders. Propensity score matching was further performed to adjust for baseline characteristics. In addition to the pre-specified variables (sex, age, and body mass index), variables significant in the univariable Cox proportional hazard regression analyses were considered for matching.

A receiver operating characteristics analysis was performed for baseline plasma BNP level to estimate the primary outcome. The patient cohort was divided into two groups by the calculated cutoff of BNP: 2.12 (equivalent to plasma BNP: 133 pg/mL).

## 3. Results

### 3.1. Baseline Characteristics

A total of 1464 patients who had undergone LAAC and were prospectively listed on the OCEAN-LAAC registry between 2019 and 2022 were eligible (Figure 1). Of them, 412 patients without baseline plasma BNP levels and 115 patients dependent on hemodialysis were excluded. Finally, 937 patients were included in this retrospective study.

Baseline characteristics are listed in Table 1. Median age was 78 (73, 83) years and 596 were men. All patients satisfied the indication of LAAC as having the risk of thromboembolic events but having difficulty in continuing anticoagulants due to several common reasons. Median CHADS_2_ score was 3 (2, 4) points, median CHA_2_DS_2_-VASc score was 5 (4, 6) points, and median HAS-BLED score was 3 (2, 3) points. In total, 440 (47%) patients had clinically diagnosed heart failure and 154 (16%) patients had left ventricular ejection fraction <50%. The common logarithm of plasma BNP level was 2.15 (1.86, 2.39) pg/mL. At baseline prior to LAAC, 65% received OAC alone and 29% received OAC and single antiplatelet therapy (SAPT). Only 2% received OAC and DAPT.

### 3.2. Comparison of Baseline Characteristics

The baseline characteristics were compared between those with high BNP and those with low BNP, which were stratified by the statistically calculated cutoff using receiver operator curve analysis of the common logarithm of plasma BNP: 2.12 (equivalent to plasma BNP: 133 pg/mL) (Table 1). Several variables were significantly different between the two groups. Patients with high BNP were older and had more advanced hypoalbuminemia, anemia, a history of heart failure, and renal impairment than their counterparts (*p* < 0.05 for all). LAA size in the high BNP group was more enlarged than its counterparts.

### 3.3. LAAC Procedure

The LAAC was carried out with two types of devices: WATCHMAN 2.5 and WATCHMAN FLX. The prevalence of device type was not significantly different between the two groups (*p* = 0.18; Table 2). Other procedure data are listed in Table 2. The implanted device was significantly larger from a statistical standpoint in the high BNP group than in its counterpart; the device differed in diameter by 1 mm, on average.

### 3.4. Peri-Procedural Outcome

Implantation success (i.e., appropriate device deployment), technical success (i.e., no device-related complications), and procedural success (i.e., no procedure-related complications) was above 95%, regardless of BNP levels (Table 3). The incidence of all peri-procedural complications was below 1%, regardless of BNP levels. The hospital stay was longer in the high BNP group (*p* < 0.001).

### 3.5. Trajectory of Medications After the Procedure

Antiplatelet and anticoagulant were adjusted after the LAAC procedure (Table 4). At index discharge, half of the participants received OAC and SAPT and the other half of them received OAC alone. During the observation period following index discharge, the prevalence of OAC prescription gradually decreased. The prevalence of these medications was mostly not significantly different between the two groups, except for those at one-year follow-up.

### 3.6. Trajectory of Echocardiography Data

Echocardiography data during short-term follow-up and at one-year follow-up are listed in Table 5. The device size was larger in the high BNP group than in its counterpart. The incidences of device compression and device protrusion were within normal range regardless of BNP levels and did not significantly differ between the two groups.

### 3.7. Primary Outcomes

During the 366 (251, 436) days of the observation period after the LAAC procedure, 148 patients encountered the primary outcome. Among the 14 variables that were considered potentially to be associated with the primary outcome, 4 variables were significant in the univariable analyses (*p* < 0.05 for all; Table 6). We did not include the history of heart failure in the multivariable analysis, given the significant multicollinearity between plasma BNP level and history of heart failure. After including five variables with *p* < 0.10 in the multivariable analysis, the common logarithm of plasma BNP level remained as an independent predictor for the primary outcome with an adjusted hazard ratio of 1.46 (95% confidence interval 1.06–2.18, *p* = 0.048, Table 6).

**Figure 2:**
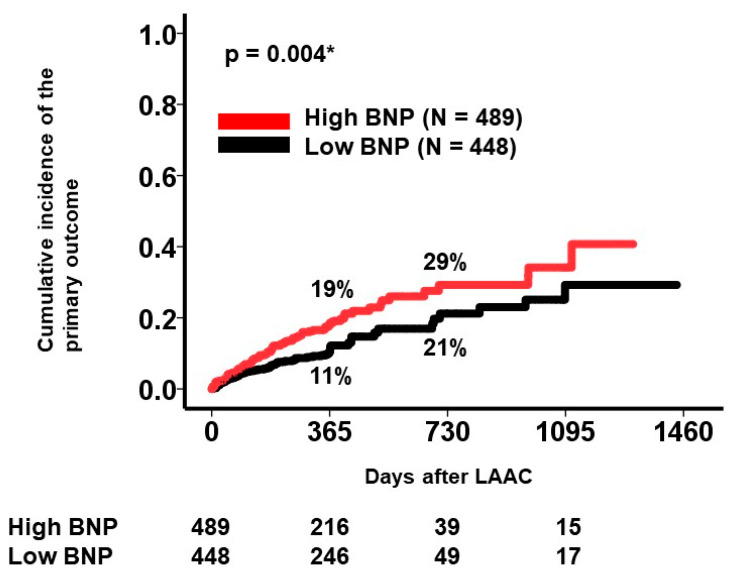
Cumulative incidence of the primary outcome stratified by the baseline plasma BNP levels. Patients were divided into two groups by the statistically calculated cutoff of baseline plasma BNP levels. The primary outcome was a composite of death, ischemic stroke, systemic embolism, device-related thrombus, gastrointestinal bleeding, hemorrhagic stroke, other major bleeding, and death. BNP, B-type natriuretic peptide; LAAC, left atrial appendage closure. * *p* < 0.05 by log-rank test.

A cutoff of the common logarithm of plasma BNP level for predicting the primary outcome was calculated as 2.12 (equivalent to plasma BNP: 133 pg/mL) with a sensitivity of 0.62 and a specificity of 0.49. The cumulative incidence of the primary outcome was significantly higher in the high BNP group (*n* = 489) compared with the low BNP group (*n* = 448) (*p* = 0.004; Figure 2). The cumulative incidence of the primary outcome remained significant between the two groups stratified by the BNP levels in a sub-group of men (*n* = 596) and women (*n* = 341), respectively (*p* = 0.006 and *p* = 0.010, respectively).

Following propensity score matching (four variables that were significant in univariable Cox proportional hazard ratio regression analyses and pre-specified sex, age, and body mass index were used for matching), 269 patients in the high BNP group and 269 patients in the low BNP group were identified. The cumulative incidence of the primary outcome remained significantly different between the two groups (2-year incidence 27% vs. 20%, *p* = 0.032).

Of the 489 patients with elevated baseline BNP levels, 290 had symptomatic heart failure. They tended to have a higher cumulative incidence of the primary outcome compared with their counterparts (i.e., sub-clinical heart failure patients), although it did not reach statistical significance (32% vs. 25% for 2-year incidence, *p* = 0.20).

### 3.8. Breakdown of the Primary Outcome

A breakdown of the primary outcomes is listed in Table 7. The incidence of ischemic stroke and death was significantly higher in the high BNP group than in their counterparts (*p* < 0.05 for both). The incidence of other events, such as gastrointestinal bleeding, did not significantly differ between the two groups (*p* > 0.05 for all).

## 4. Discussion

In this retrospective analysis using prospectively collected multi-center large-scale OCEAN-LAAC registry data, we investigated the impact of higher baseline plasma BNP level on the occurrence of death or stroke/bleeding events in patients with non-valvular atrial fibrillation receiving LAAC. Among the LAAC candidates, individuals with higher plasma BNP had more advanced anemia, hypoalbuminemia, renal impairment, and more enlarged LAA than their counterparts. The LAAC was successfully implanted with a few peri-procedural complications regardless of the levels of plasma BNP. Following LAAC, the types and prescription rates of antithrombotic medications were comparable regardless of the levels of plasma BNP. The echocardiographic findings during the observation period were comparable irrespective of the levels of plasma BNP, except for the larger device size in patients with high BNP. Of note, the incidence of device-related thrombus was approximately 1–2% in both groups. A higher baseline plasma BNP level was independently associated with death and stroke.

### 4.1. Safety of LAAC in Patients with Elevated BNP Levels

Previous literature demonstrated the implantation success of LAAC has exceeded 95%, given the institutional learning curve, device innovation, patient selection, pre-procedural image assessment, improved peri-procedural management, appropriate post-LAAC anti-thrombotic medication therapy, and careful post-operative monitoring following index discharge [26]. Nevertheless, few studies demonstrated the safety of contemporary LAAC therapy in patients with heart failure. The presence of heart failure, particularly the elevated plasma BNP that indicates incremental increases in intra-cardiac filling pressures, leads to peri-procedural worsening of clinical status during various invasive procedures [27].

We demonstrated that implantation, technical, and procedural successes were at satisfactory levels of above 90%, irrespective of the presence of elevated BNP levels. This was despite the higher prevalence of multiple comorbidities and more progressed end-organ dysfunction in those classified with higher BNP levels. The incidence of peri-procedural complications was statistically comparable irrespective of the plasma BNP levels, except for slightly longer lengths of stay in patients with elevated BNP levels.

Previous literature has also described comparable in-hospital mortality and cardiac complication rates in the LAAC candidates irrespective of the presence of heart failure, whereas the incidence of non-cardiac complications was higher in individuals with heart failure, such as acute kidney injury and respiratory failure [28]. Another study also showed comparable implant success and peri-procedural complication rates between those with and without heart failure [29]. However, the odds ratio of post-LAAC cardiovascular death was 3.64 in those with a history of heart failure vs. those without heart failure. Other studies also support the association between heart failure and worse prognosis after LAAC [30,31]. Given such evidence, together with our findings, an elevated plasma BNP level alone does not preclude the indication of LAAC. Nevertheless, estimated survival times may differ and should be discussed with patients and their relatives before LAAC in individuals with elevated plasma BNP levels.

### 4.2. Efficacy of LAAC in Patients with Elevated BNP Levels

In the present study, an elevated baseline plasma BNP level, a surrogate of the presence of heart failure, was associated with the occurrence of ischemic stroke after LAAC. Heart failure is a well-known risk factor for stroke likely due to chamber dilation and worsened ventricular contractility, which in totality increase the risk of blood stasis and clot formation. CHADS_2_ score and CHA_2_DS_2_-VASc score include the presence of heart failure as a component that increases the risk of stroke in patients with non-valvular atrial fibrillation. Left ventricular dysfunction is one of the risk factors of device-related thrombosis after LAAC [32] although it did not reach statistical significance in the present study, probably due to small event numbers.

One of the underlying diseases that trigger heart failure is chronic inflammation, which also exerts thrombus formation. Aggressive use of diuretics to manage congestive heart failure induces intravascular hypovolemia and increases the risk of thrombosis. Impaired cardiac contractility (both ventricular and atrial) induces intra-cardiac turbulence and increases the risk of thrombus formation.

### 4.3. BNP Level and Atrial Abnormality

We focused on the level of plasma BNP, instead of the concomitant presence of heart failure, to more granularly stratify risk. Recent literature identified the level of plasma BNP as an independent risk factor of thromboembolic events in patients with non-valvular atrial fibrillation, irrespective of the spectrum of ejection fraction [33,34]. In the recently introduced ABC-stroke score, the presence of heart failure is replaced with the level of NT-pro BNP [35]. Symptoms of heart failure overlap with those of atrial fibrillation, and it is sometimes challenging to correctly identify the concomitant presence of heart failure in patients with atrial fibrillation [22].

Furthermore, the presence of paroxysmal atrial fibrillation can be surveyed among potential individuals with elevated BNP levels, which represents atrial abnormality [36]. The elevated plasma BNP level directly represents atrial abnormality which may increase the risk of thrombus formation. The electrical expression of atrial abnormality is atrial fibrillation, the endothelial expression of atrial abnormality is thrombus formation, and the hemodynamic expression of atrial abnormality is heart failure. These are rationales for why we utilized plasma BNP levels as an independent variable.

### 4.4. Elevated BNP Level and Adverse Events

Previous studies have demonstrated a significant association between elevated BNP levels and either low LAA flow velocity or the presence of LAA thrombus, irrespective of the spectrum of ejection fraction [37]. In the present study, the size of LAA was larger in patients with high BNP. However, the clinical impact of LAA enlargement remains uncertain. The LAA is well known as an endocrinological organ, especially for natriuretic peptides [38]. The findings of the LAA in relation to BNP levels in this study could potentially be explained by the theory that LAA enlargement leads to an increase in BNP levels.

Another potential mechanism for LAA enlargement involves hemodynamic compensation for elevated left ventricular end-diastolic pressure. Regardless, the LAAC might have been insufficient in the patients with high BNP. However, the incidence of device-related thrombus was comparable regardless of the levels of BNP.

Enlargement of the LAA may serve as a surrogate marker for left atrial endocardial dysfunction, potentially facilitating thrombus formation within the extra-LAA space. This theory is substantiated by a previous work, which demonstrated endocardial thickening characterized by an accumulation of fibrous and elastic tissue in enlarged LAA [38]. Interestingly, several recent studies have suggested an association between LAAC and adverse hemodynamic events, particularly an increased risk of worsening heart failure [30]. As this association is thought to be due to a reduction in left atrial reservoir function, the relationship between LAAC and hemodynamic derangements might be associated with an increased risk of all-cause mortality as seen in the present study. The event rates overall were not high enough to determine a more robust association but are hypothesis generating.

Moreover, patients with elevated BNP had a higher prevalence of coronary artery disease. Such patients might have had more atherosclerotic cerebral infarction. Of note, it may be challenging to distinguish atherosclerotic cerebral infarction and thromboembolic one.

### 4.5. Clinical Implication of Our Findings

Our findings can be useful for risk stratification before LAAC and shared-decision making, specifically as the presence of heart failure may be associated with higher adverse event rates post-procedure. We should emphasize to the patients and their relatives that LAAC does not completely prohibit stroke/bleeding events. The efficacy of LAAC may decrease in patients with elevated plasma BNP levels. Patients should undergo LAAC after understanding the limitations of the LAAC, particularly in individuals with elevated BNP levels. Careful monitoring might be required for individuals with elevated plasma BNP levels after LAAC to survey early signs of adverse events.

Some centers with increased procedural experience may opt to withdraw anti-thrombotic agents earlier than that point recommended following the initial commercial implants of the device [39]. However, anti-thrombotic regimens may need to be re-considered in terms of strength and post-procedure duration in patients with higher levels of plasma BNP. For example, the timing to terminate anticoagulants may be prolonged following LAAC in patients with elevated plasma BNP levels, given their high risk for stroke. Further studies are warranted to validate such a modified anti-thrombotic regimen in individuals with elevated BNP levels.

Conversely, a history of bleeding was another independent risk factor of stroke/bleeding events. Several factors, including age, female sex, and renal dysfunction, are recognized overlapping risk factors for both stroke and bleeding [40]. Thus, subjects identified in the present study with certain risk factors may have a low safety margin regarding both stroke and bleeding. Further studies are needed to better cultivate an optimal anti-thrombotic regimen [41].

## 5. Limitations

Several limitations of the present study should be addressed. We tried to include many potential variables in the time-to-event analyses, but other uninvestigated confounders might have existed. Given the large-scale registry, we could not collect comprehensive clinical data elements, including heart failure medication. It is uncertain whether aggressive intervention for heart failure by titrating heart failure medications may improve clinical outcomes following LAAC. Given the nature of the observational study, we could not investigate the underlying causality between the elevated plasma BNP levels and stroke/bleeding events. We should emphasize that we focused on the plasma BNP levels. An elevated plasma BNP level does not simply represent the presence of heart failure. We excluded patients without plasma BNP values, and cannot deny the presence of selection bias. We compared the baseline characteristics of the present study vs. those of the excluded cohort due to missing BNP values. There were no significant differences in the baseline characteristics between the two groups (*p* > 0.05 for all). We want to emphasize that plasma BNP levels may not be mandatorily measured before LAAC and according to our findings, we strongly recommend measuring plasma BNP levels before LAAC, irrespective of the presence/history of heart failure for risk stratification, as we demonstrated here.

## 6. Conclusions

Using prospectively collected large-scale multi-center Japanese registry data, we demonstrated that a baseline higher plasma BNP level was independently associated with a higher incidence of stroke and mortality after LAAC. Further studies are warranted for the optimal therapeutic strategy for LAAC candidates with elevated baseline plasma BNP levels.

## Figures and Tables

**Table 1:** Baseline characteristics.

	Total (*n* = 937)	High BNP (*n* = 489)	Low BNP (*n* = 448)	*p*-Value
Demographics				
Age, years	78 (73, 83)	80 (75, 84)	77 (72, 81)	<0.001 *
Men	596 (64%)	292 (60%)	196 (44%)	0.006 *
Body mass index, kg/m^2^	23.1 (20.7, 25.5)	22.9 (20.0, 25.4)	23.4 (21.4, 25.7)	0.003 *
Systolic blood pressure, mmHg	125 (112, 138)	124 (110, 138)	126 (114, 138)	0.13
Pulse rate, bpm	70 (62, 79)	70 (61, 79)	71 (62, 79)	0.96
Rhythm: sinus/atrial fibrillation/pacemaker	318/550/69	92/352/45	226/198/24	<0.001 *
Comorbidity				
Hypertension	680 (73%)	353 (72%)	327 (73%)	0.95
Diabetes mellitus	309 (33%)	167 (34%)	142 (32%)	0.23
Dyslipidemia	384 (41%)	204 (42%)	180 (40%)	0.34
Heart failure	440 (47%)	290 (59%)	150 (33%)	<0.001 *
Coronary artery disease	418 (45%)	246 (50%)	172 (38%)	<0.001 *
Pacemaker implantation	107 (11%)	65 (13%)	42 (9%)	0.037 *
Peripheral artery disease	112 (12%)	80 (16%)	32 (7%)	<0.001 *
History of major bleeding	578 (62%)	284 (58%)	294 (66%)	0.010 *
History of ischemic stroke	376 (40%)	193 (39%)	183 (41%)	0.36
History of hemorrhagic stroke	120 (13%)	58 (12%)	62 (14%)	0.21
Hemodialysis dependence	0 (0%)	0 (0%)	0 (0%)	-
Scores				
CHADS_2_ score	3 (2, 4)	3 (3, 4)	3 (2, 4)	<0.001 *
CHA_2_DS_2_-VASc score	5 (4, 6)	5 (4, 6)	5 (4, 6)	<0.001 *
HAS-BLED score	3 (2, 3)	3 (2, 4)	3 (2, 3)	0.058
Laboratory data				
Serum albumin, g/dL	3.9 (3.6, 4.1)	3.8 (3.6, 4.1)	3.9 (3.7, 4.2)	<0.001 *
Hemoglobin, g/dL	12.4 (10.9, 13.8)	11.9 (10.4, 13.5)	12.8 (11.5, 14.0)	<0.001 *
Serum creatinine, mg/dL	1.0 (0.8, 1.3)	1.1 (0.9, 1.5)	0.9 (0.8, 1.1)	<0.001 *
Common logarithm of plasma BNP, pg/mL	2.15 (1.86, 2.39)	2.37 (2.24, 2.57)	1.83 (1.60, 2.00)	<0.001 *
PT-INR	1.3 (1.1, 1.5)	1.3 (1.1, 1.6)	1.2 (1.1, 1.5)	<0.001 *
TTE data				
LVDd, mm	46 (42, 51)	47 (43, 52)	45 (42, 50)	0.023 *
LVEF, %	60 (53, 65)	60 (51, 66)	62 (58, 68)	<0.001 *
LVEF < 50%	154 (16%)	109 (22%)	45 (10%)	<0.001 *
LA diameter, mm	44 (40, 50)	46 (41, 52)	43 (38, 49)	<0.001 *
TRPG, mmHg	25 (20, 31)	27 (22, 32)	23 (19, 29)	<0.001 *
MR moderate or greater	107 (11%)	72 (15%)	35 (8%)	0.001 *
TR moderate or greater	166 (18%)	121 (25%)	45 (10%)	<0.001 *
TEE data				
LAA ostium diameter, mm				
0 degree	22 (20, 25)	23 (20, 25)	22 (19, 24)	<0.001 *
45 degrees	21 (19, 23)	21 (19, 23)	20 (18, 23)	0.002 *
90 degrees	21 (19, 24)	22 (20, 24)	21 (18, 24)	0.001 *
135 degrees	23 (20, 26)	24 (21, 25)	22 (19, 25)	<0.001 *
LAA depth, mm				
0 degree	26 (21, 31)	27 (21, 33)	25 (20, 29)	<0.001 *
45 degrees	26 (21, 30)	27 (22, 32)	24 (18, 29)	<0.001 *
90 degrees	26 (20, 31)	27 (22, 32)	24 (19, 30)	<0.001 *
135 degrees	25 (21, 30)	27 (23, 31)	24 (19, 28)	<0.001 *
LAA filling flow velocity, cm/sec	27 (18, 44)	21 (16, 38)	34 (19, 51)	<0.001 *
LAA emptying flow velocity, cm/sec	24 (17, 43)	21 (16, 34)	32 (17, 52)	<0.001 *
Complex aortic plaque	28 (3%)	17 (3%)	11 (2%)	0.21
Medications				
OAC + DAPT	20 (2%)	12 (2%)	8 (2%)	0.32
OAC + SAPT	269 (29%)	153 (31%)	116 (26%)	0.040 *
OAC alone	608 (65%)	299 (61%)	309 (69%)	0.007 *
DAPT alone	6 (1%)	4 (1%)	2 (0%)	0.48
SAPT alone	7 (1%)	6 (1%)	1 (0%)	0.075
None	8 (1%)	6 (1%)	2 (0%)	0.20

Continuous variables are stated as median (25% interquartile, 75% interquartile) and compared between the two groups by the Mann–Whitney U test. Categorical variables are stated as numbers (percentage) and compared between the two groups by Chi-square test or Fischer’s exact test. BNP, B-type natriuretic peptide; PT-INR, prothrombin time with international normalized ratio; TTE, transthoracic echocardiography; LVDd, left ventricular end-diastolic diameter; LVEF, left ventricular ejection fraction; LA, left atrium; TRPG, tricuspid regurgitation pressure gradient; MR, mitral regurgitation; TR, tricuspid regurgitation; TEE, transesophageal echocardiography; LAA, left atrial appendage; OAC, oral anticoagulation; DAPT, dual antiplatelet therapy; SAPT, single antiplatelet therapy. * *p* < 0.05.

**Table 2:** Procedure data.

	High BNP (*n* = 489)	Low BNP (*n* = 448)	*p*-Value
Implanted device, 2.5/FLX	183/306	154/294	0.18
General data			
Anesthesia time, min	93 (77, 111)	93 (77, 112)	0.91
Procedure time, min	45 (35, 60)	46 (37, 61)	0.78
Fluoroscopy duration, min	12 (9, 17)	12 (9, 16)	0.20
Contrast volume, mL	50 (30, 66)	55 (37, 80)	<0.001 *
Implanted device size, mm	31 (27, 33)	30 (27, 31)	<0.001 *
Maximum device diameter, mm			
0 degree	25 (22, 27)	24 (22, 27)	<0.001 *
45 degrees	25 (23, 27)	24 (21, 27)	<0.001 *
90 degrees	25 (23, 27)	24 (22, 27)	0.001 *
135 degrees	25 (23, 28)	24 (22, 27)	<0.001 *
Device compression, %			
0 degree	17 (13, 21)	16 (13, 21)	0.47
45 degrees	17 (13, 21)	17 (13, 21)	0.52
90 degrees	16 (13, 20)	16 (13, 20)	0.081
135 degrees	15 (13, 19)	15 (13, 20)	0.84
Device protrusion, mm			
0 degree	3.0 (0, 6.0)	3.5 (0, 6.0)	0.28
45 degrees	3.2 (0, 5.8)	3.2 (0, 5.0)	0.49
90 degrees	5.1 (2.0, 7.9)	5.0 (2.0, 8.0)	0.70
135 degrees	6.0 (3.1, 8.3)	6.0 (3.7, 9.0)	0.58
Deep device implantation	5 (1%)	10 (2%)	0.11
Residual trabeculation	30 (6%)	27 (6%)	0.50

Continuous variables are stated as median (25% interquartile, 75% interquartile) and compared between the two groups by the Mann–Whitney U test. Categorical variables are stated as numbers (percentage) and compared between the two groups by Chi-square test or Fischer’s exact test. BNP, B-type natriuretic peptide. * *p* < 0.05.

**Table 3:** Peri-procedural outcome.

	High BNP (*n* = 489)	Low BNP (*n* = 448)	*p*-Value
Implantation outcome			
Implantation success	476 (98%)	438 (98%)	0.42
Technical success	476 (97%)	436 (97%)	0.57
Procedural success	460 (94%)	428 (96%)	0.20
Peri-procedural complications			
Access site complication	4 (1%)	3 (1%)	0.79
TEE-related complication	2 (0%)	0 (0%)	0.18
Acute kidney injury	2 (0%)	0 (0%)	0.18
Device embolization	0 (0%)	0 (0%)	-
Infectious endocarditis	0 (0%)	0 (0%)	-
Any strokes	0 (0%)	0 (0%)	-
Major bleedings	2 (0%)	2 (0%)	0.93
Length of hospital stay, days	5 (4, 6)	4 (4, 6)	<0.001 *

Continuous variables are stated as median (25% interquartile, 75% interquartile) and compared between the two groups by the Mann–Whitney U test. Categorical variables are stated as numbers (percentage) and compared between the two groups by Chi-square test or Fischer’s exact test. BNP, B-type natriuretic peptide. * *p* < 0.05.

**Table 4:** Trajectory of medication after the procedure.

	High BNP (*n* = 489)	Low BNP (*n* = 447)	*p*-Value
Discharge			
OAC + DAPT (3 medications)	11 (2%)	5 (1%)	0.14
OAC + SAPT (2 medications)	236 (48%)	221 (49%)	0.40
OAC alone (1 medication)	228 (47%)	212 (47%)	0.44
DAPT alone (2 medications)	2 (0%)	1 (0%)	0.62
SAPT alone (1 medication)	4 (1%)	2 (0%)	0.48
None (0 medication)	3 (0%)	11 (2%)	0.36
Three-month follow-up			
OAC + DAPT (3 medications)	2 (0%)	2 (0%)	0.93
OAC + SAPT (2 medications)	53 (11%)	40 (9%)	0.19
OAC alone (1 medication)	107 (22%)	95 (21%)	0.43
DAPT alone (2 medications)	141 (29%)	139 (31%)	0.25
SAPT alone (1 medication)	144 (29%)	140 (31%)	0.30
None (0 medication)	11 (2%)	18 (4%)	0.085
One-year follow-up			
OAC + DAPT (3 medications)	0 (0%)	0 (0%)	-
OAC + SAPT (2 medications)	17 (3%)	9 (2%)	0.12
OAC alone (1 medication)	39 (8%)	51 (11%)	0.049 *
DAPT alone (2 medications)	13 (3%)	15 (3%)	0.33
SAPT alone (1 medication)	229 (47%)	235 (53%)	0.049 *
None (0 medication)	20 (4%)	33 (7%)	0.021 *

Categorical variables are stated as numbers (percentage) and compared between the two groups by Chi-square test or Fischer’s exact test. BNP, B-type natriuretic peptide; OAC, oral anticoagulation; DAPT, dual antiplatelet therapy; SAPT, single antiplatelet therapy. * *p* < 0.05.

**Table 5:** Trajectory of echocardiography data during a one-year observation period.

	High BNP (*n* = 489)	Low BNP (*n* = 447)	*p*-Value
Short-term follow-up			
Maximum device diameter, mm			
0 degree	26 (24, 28)	24 (22, 27)	0.001 *
45 degrees	25 (23, 28)	24 (22, 27)	0.001 *
90 degrees	26 (24, 28)	24 (22, 27)	0.012 *
135 degrees	26 (24, 28)	25 (22, 27)	0.002 *
Device compression, %			
0 degree	14 (10, 18)	14 (11, 17)	0.49
45 degrees	15 (12, 19)	14 (11, 19)	0.43
90 degrees	15 (11, 18)	13 (10, 19)	0.002 *
135 degrees	14 (11, 18)	13 (10, 17)	0.077
Device protrusion, mm			
0 degree	3.5 (0, 6.0)	4.1 (0, 6.0)	0.30
45 degrees	4.0 (0, 6.1)	3.9 (0, 6.0)	0.73
90 degrees	4.8 (0, 7.0)	4.1 (0, 6.4)	0.33
135 degrees	5.4 (1.6, 7.7)	4.9 (2.2, 6.8)	0.38
Deep device implantation	6 (1%)	4 (1%)	0.83
Device-related thrombus	4 (1%)	2 (0%)	0.63
One-year follow-up			
Maximum device diameter, mm			
0 degree	26 (24, 28)	25 (22, 27)	0.042 *
45 degrees	26 (23, 28)	24 (22, 27)	0.074
90 degrees	25 (24, 28)	24 (22, 28)	0.032 *
135 degrees	26 (24, 28)	25 (23, 28)	0.13
Device compression, %			
0 degree	15 (11, 19)	14 (11, 18)	0.020 *
45 degrees	17 (12, 21)	15 (10, 19)	0.008 *
90 degrees	16 (12, 20)	13 (11, 19)	0.032 *
135 degrees	14 (11, 18)	12 (9, 16)	<0.001 *
Device protrusion, mm			
0 degree	3.0 (0, 5.5)	1.5 (0, 5.0)	0.19
45 degrees	3.9 (0, 6.0)	3.2 (0, 5.2)	0.30
90 degrees	4.1 (0, 7.5)	3.9 (0, 6.0)	0.38
135 degrees	3.6 (0, 5.6)	3.0 (0, 5.8)	0.75
Deep device implantation	6 (1%)	2 (0%)	0.18
Device-related thrombus	11 (2%)	6 (1%)	0.33

Continuous variables are stated as median (25% interquartile, 75% interquartile) and compared between the two groups by the Mann–Whitney U test. Categorical variables are stated as numbers (percentage) and compared between the two groups by Chi-square test or Fischer’s exact test. BNP, B-type natriuretic peptide. * *p* < 0.05.

**Table 6:** Baseline characteristics associated with the primary outcome.

	Univariable Analysis		Multivariable Analysis	
	Hazard Ratio (95% CI)	*p*-Value	Hazard Ratio (95% CI)	*p*-Value
Age, years	0.99 (0.98–1.02)	0.84		
Hypertension	0.94 (0.69–1.28)	0.68		
Diabetes mellitus	1.15 (0.82–1.61)	0.41		
Heart failure	1.85 (1.33–2.58)	<0.001 *	-	-
History of major bleeding	1.48 (1.04–2.11)	0.030 *	1.48 (1.02–2.14)	0.037 *
History of ischemic stroke	0.74 (0.52–1.04)	0.082	0.85 (0.60–1.20)	0.35
History of hemorrhagic stroke	1.16 (0.73–1.84)	0.53		
CHADS_2_ score	1.05 (0.93–1.19)	0.40		
CHA_2_DS_2_-VASc score	1.01 (0.91–1.13)	0.79		
HAS-BLED score	1.01 (0.85–1.19)	0.95		
Serum creatinine, mg/dL	1.31 (1.15–1.50)	<0.001 *	1.28 (1.09–1.51)	0.002 *
Common logarithm of plasma BNP, pg/mL	1.65 (1.13–2.42)	0.009 *	1.46 (1.06–2.18)	0.043 *
Implanted device FLX vs. 2.5	0.86 (0.61–1.21)	0.37		
Number of medications	0.78 (0.58–1.04)	0.093	0.82 (0, 0.61–1.10)	0.18

Cox proportional hazard ratio regression analyses were performed to investigate the association between the potential variables and the primary outcome. The primary outcome was defined as a composite of death, major stroke events, and major bleeding events. Variables that were potentially considered to be associated with the primary outcome were included in the univariable analyses. Variables with *p* < 0.10 in the univariable analyses were included in the multivariable analysis. CI, confidence interval; BNP, B-type natriuretic peptide. * *p* < 0.05.

**Table 7:** Breakdown of the primary outcomes.

	High BNP (*n* = 489)	Low BNP (*n* = 447)	*p*-Value
Ischemic stroke	20 (4%)	7 (2%)	0.016 *
Systemic embolism	2 (0%)	1 (0%)	0.62
Device-related thrombus	15 (3%)	14 (3%)	0.55
Gastrointestinal bleeding	22 (4%)	27 (6%)	0.18
Hemorrhagic stroke	4 (1%)	5 (1%)	0.64
Other major bleeding events	8 (2%)	11 (2%)	0.37
Death	44 (9%)	15 (3%)	<0.001 *

The numbers of patients who encountered each event are stated. Categorical variables are stated as numbers (percentage) and compared between the two groups by Chi-square test or Fischer’s exact test. BNP, B-type natriuretic peptide; OAC, oral anticoagulation; DAPT, dual antiplatelet therapy; SAPT, single antiplatelet therapy. * *p* < 0.05.

## Data Availability

The data presented in this study are available on request from the corresponding author. Please specify the reason for restriction, e.g., the data are not publicly available due to privacy or ethical restrictions.

## Supplementary Materials

The following supporting information can be downloaded at: https://www.mdpi.com/article/10.3390/jcm13206232/s1. Supplementary Table S1. Participating sites and investigators
