# Supplementary material for: Correlations Between Plasma BNP Level and Risk of Thrombotic-Hemorrhagic Events After Left Atrial Appendage Closure
Source: J Clin Med. 2024 Oct 18;13(20):6232. doi: 10.3390/jcm13206232 (PMC11508434; doi:10.3390/jcm13206232)
Supplement: Supplementary file 1 [file jcm-13-06232-s001.zip › jcm-3205804-supplementary.pdf]

Supplementary Table S1. Participating sites and investigators

Site	Department	Investigator	Role	Location
Keio University School of Medicine	Department of Cardiology	Kentaro Hayashida	Site PI	Tokyo
Toyohashi Heart Center	Department of Cardiology	Masanori Yamamoto	Site PI	Aichi
Nagoya Heart Center	Department of Cardiology	Masanori Yamamoto	Site PI	Aichi
Gifu Heart Center	Department of Cardiology	Masanori Yamamoto	Site PI	Gifu
Kokura Memorial Hospital	Department of Cardiology	Shinichi Shirai	Site PI	Fukuoka
Teikyo University School of Medicine	Department of Cardiology	Yusuke Watanabe	Site PI	Tokyo
New Tokyo Hospital	Department of Cardiology	Toru Naganuma	Site PI	Chiba
Sendai Kousei Hospital	Department of Cardiology	Masaki Nakashima	Site PI	Miyagi
Shonan Kamakura General Hospital	Department of Cardiology	Shingo Mizuno	Site PI	Kanagawa
Kinki University School of Medicine	Department of Cardiology	Kazuki Mizutani	Site PI	Osaka
Toyama University Hospital	Department of Cardiology	Hiroshi Ueno	Site PI	Toyama
Tokai University School of Medicine	Department of Cardiology	Yohei Ohno	Site PI	Kanagawa
St. Marianna University School of Medicine	Department of Cardiology	Masaki Izumo	Site PI	Kanagawa
Sapporo East Tokushukai Hospital	Department of Cardiology	Tomoyuki Tani	Site PI	Hokkaido
Saiseikai Kumamoto Hospital	Department of Cardiology	Hideharu Okamatsu	Site PI	Kumamoto
Kurashiki Central Hospital	Department of Cardiology	Shunsuke Kubo	Site PI	Okayama
Mitsui Memorial Hospital	Division of Cardiology	Masahiko Asami	Site PI	Tokyo
Sakakibara Heart Institute	Department of Cardiology	Mike Saji	Site PI	Tokyo
